# Linking genome variants to disease: scalable approaches to test the functional impact of human mutations

**DOI:** 10.1093/hmg/ddab219

**Published:** 2021-08-02

**Authors:** Gregory M Findlay

**Affiliations:** The Francis Crick Institute, The Genome Function Laboratory, London NW1 1AT, UK

## Abstract

The application of genomics to medicine has accelerated the discovery of mutations underlying disease and has enhanced our knowledge of the molecular underpinnings of diverse pathologies. As the amount of human genetic material queried via sequencing has grown exponentially in recent years, so too has the number of rare variants observed. Despite progress, our ability to distinguish which rare variants have clinical significance remains limited. Over the last decade, however, powerful experimental approaches have emerged to characterize variant effects orders of magnitude faster than before. Fueled by improved DNA synthesis and sequencing and, more recently, by CRISPR/Cas9 genome editing, multiplex functional assays provide a means of generating variant effect data in wide-ranging experimental systems. Here, I review recent applications of multiplex assays that link human variants to disease phenotypes and I describe emerging strategies that will enhance their clinical utility in coming years.

## Introduction: The Challenge of Going from Variant to Function

Millions of human exomes and genomes have now been sequenced, yet we have only observed a small fraction of the rare variants in people alive today. Estimates of *de novo* mutation rates suggest that every single nucleotide variant (SNV) compatible with life occurs at least once per generation (1). In the genome aggregation database (gnomAD), comprising exomes and genomes from ~141 000 individuals, the majority of observed variants occur in exactly one individual and only 11.5% of possible synonymous SNVs occur at all (2). Improved sampling of genetically diverse populations will undoubtedly reveal new variants associated with phenotypes (3–5) but will also yield more rare variants whose phenotypic consequences are unknown. Similar to germline variants, large numbers of somatic mutations have been observed across cancer genomes (6,7). A small fraction occurs repeatedly, yet vastly more are unique variants with unknown effects on disease.

Through approaches ranging from direct-to-consumer genetic testing and liquid biopsies to whole-exome and whole-genome sequencing, more patients than ever before are receiving genetic test results (8). The value of identifying a causal germline variant has been well established for monogenic diseases (9–11). Furthermore, targeted therapies available to treat genetic diseases and cancers are giving clinicians the means to capitalize on the knowledge of variant effect more than ever before (12–16).

Yet, the translational potential of genomics remains limited largely by our inability to predict which variants observed in patients influence actionable phenotypes. For coding variants in genes commonly sequenced, this problem is manifest in hundreds of thousands of variants of uncertain significance (VUS) in databases such as ClinVar (17). These are often missense or splice variants that may alter a gene’s function in one of several ways (e.g*.* loss-of-function and gain-of-function), or have no discernible effect at all. In non-coding sequence, genome-wide association studies (GWAS) have linked thousands of loci to disease (18–20), yet pinpointing causal variants and discovering the precise mechanisms through which they act remain major bottlenecks to discovery (21). Missing heritability estimates suggest that rare variants of large effect are often missed by current ascertainment practices (22,23). This is further supported by less-than-predicted diagnostic yields when genetic testing is performed for many conditions (24,25).

The challenge of rare variant interpretation stems from an incomplete molecular accounting of how changes to deoxyribonucleic acid (DNA) sequence alter function on the molecular, cellular and organismal levels. Classical genetics approaches enable variant–phenotype associations without requiring knowledge of mechanism, but despite growing cohorts and genomic coverage, they still lack statistical power for most rare variants (26). Computational models that leverage, for instance, sequence conservation (27–29), epigenetic profiling (30,31) and/or biochemical properties of proteins (32,33) have improved, but they do not display the accuracy required for clinical variant classification without additional evidence.

Functional assays allow researchers to assess variant effects in isolation—e.g*.* determining how a missense mutation alters enzymatic activity, or how a promoter variant impacts gene expression. It is difficult to develop assays that guarantee high clinical impact, however, largely because variants may exert phenotypic effects through myriad molecular mechanisms. Furthermore, the incomplete penetrance, variable expressivity and pleiotropy observed across genetic disease highlight how complex making clinical predictions from molecular phenotypes can be. Despite these challenges, if a given element is linked to disease, functional data can be highly informative. Accordingly, American College of Medical Genetics and Genomics (ACMG) guidelines allow well-validated experimental data to serve as strong evidence of pathogenicity (34).

Until recently, efforts to classify variants experimentally have scaled poorly. The vast majority of variants observed in patients—even many known to be associated with human phenotypes—have never been tested in a laboratory setting. This may be starting to change, however, with the introduction of functional assays to measure variant effects at scale (35–37).

In this review, I describe how multiplex assays are enhancing our understanding of variant function in disease, with a focus on emerging strategies for increasing clinical impact. The challenge of variant interpretation often requires multiple assays that measure different molecular and cellular phenotypes to more fully unravel disease mechanisms. Relatedly, advances in genome editing are allowing variants to be assayed in their endogenous context more easily. Seamless integration of assay data with catalogs of human variation linked to phenotypic data will allow researchers to rapidly relate experimental findings to clinical significance. Therefore, as multiplex assays continue to shed light on the mechanisms underlying variant-phenotype associations, it follows that soon many more rare variants will become actionable, leading to tangible benefits for greater numbers of patients.

## Multiplex assays: measuring variant effects with deep sequencing

**Figure 1:**
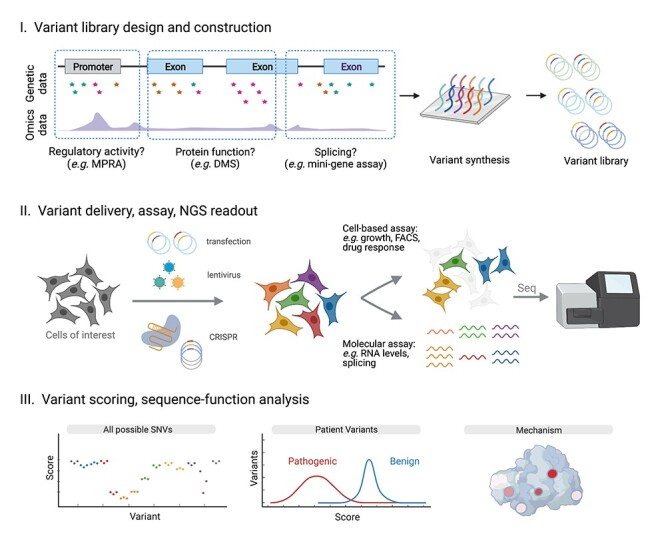
Principles of multiplex assays for interrogating human variant effects. Regions for mutagenesis are chosen from the genome with consideration of variants associated with disease and various omics data sets. In experimental design (Step I), variant alleles are cloned into an assay-specific construct, such as a reporter vector (MPRA), expression constructs (DMS), minigene cassettes (splice assays) or constructs to facilitate genome editing (SGE). Variants are then introduced to cells to create a diverse population in which each variant is present in many cells (Step II). Cell-based and molecular assays compatible with NGS are used to readout the effect of each variant in the pooled population. In analysis (Step III), sequencing counts are used to assign variants scores that can be compared to established pathogenic and benign variants. Integrated analysis with other sources of data (e.g*.* protein structure) can lead to mechanistic insights.

Multiplex assays serve to reveal how each of many DNA sequences alter biological function. Sometimes broadly referred to as multiplex assays of variant effect (MAVEs) (37), these methods include deep mutational scanning (DMS), massively parallel reporter assays (MPRAs) and saturation genome editing (SGE), among others (35,38–40) (Fig. 1).

What makes multiplex assays highly scalable is that variants are engineered and tested in a pooled format, drastically reducing cost and minimizing sample processing. This is possible because next-generation sequencing (NGS) is used in a quantitative fashion to report on the functional effects of each variant in a pool—i.e*.* to ‘readout’ the assay. As standard NGS protocols can provide billions of sequencing reads (41), sequencing-based readouts have the statistical power to make hundreds of thousands of quantitative measurements of variant effect.

Since multiplex assays were first demonstrated over a decade ago, methods for engineering variant libraries and quantifying results with NGS have matured substantially (37,42). There are robust strategies to avoid experimental bottlenecks and analysis frameworks to faithfully extrapolate variant effect measurements from sequencing data (43–47). This has made it possible for small teams of scientists to quickly test thousands to hundreds of thousands of variants with minimal noise, though care must be taken to preserve data quality.

Because multiplex assays rely on NGS to measure variant effects, relatively simple assays have predominated to date. Common approaches to link variants to their effects rely on selection of phenotypes, such as cell growth (e.g*.* gene essentiality and drug resistance) (40,48–52), fluorescence (e.g*.* fluorescence-activated cell sorting (FACS)-based on target protein abundance or reporter expression) (53–56) or biochemical properties (57). Pools of cells are sampled corresponding to different timepoints, treatments or phenotypes, and sequencing of each pool enables comparison of variant frequencies across the experiment. For instance, if a variant becomes highly abundant after a drug treatment, we may infer it confers resistance. A common strategy for assays that look at regulatory element function or splicing is to assess transcript abundances through targeted RNA sequencing, often using molecular barcoding. A distinct advantage of multiplex assays is that experiments are internally controlled via inclusion of sequences with known effects, facilitating systematic comparisons of all variants in relation to those with established phenotypes.

## Developing multiplex assays with high clinical relevance

The most immediate clinical impact of multiplex assays is being made via their application to coding sequences, largely because so many missense variants of unknown function have been encountered clinically. The groundwork for studying missense variants at scale was established in 2010. Fowler *et al.* (57) introduced deep mutational scanning to interrogate >600 000 variants in the human WW domain using phage display. Concurrently, Ernst *et al.* (58) used a similar strategy to test PDZ domain variants. Since then, over a million variants across dozens of different proteins have been engineered and assayed to examine effects on processes including protein stability, enzymatic activity, binding interactions, folding and structure, allostery and many more (51,55,59–65). The nuances of different assays have been reviewed elsewhere (42), and a thorough listing is available online (see https://www.mavedb.org) (66).

Recent deployments of functional assays to study protein variants have proven to be highly accurate at predicting pathogenicity when results are benchmarked to clinically established annotations. A yeast complementation assay was used to create a variant-effect map for *CBS*, the gene underlying classical homocystinuria (67). In addition to predicting pathogenic variants more accurately than computational models, the authors show that the degree of assay impairment correlates with the age of disease onset and severity in patients. Likewise, a study of >14 000 amyloid beta variants’ effects on aggregation enabled accurate identification of all 12 familial Alzheimer’s variants known to act dominantly despite the assay being performed in yeast (68). The first DMS of *MSH2*, a mismatch repair gene underlying Lynch syndrome in which >2000 VUS have been reported, achieved over 95% concordance with prior clinical interpretations of missense variants using 6-thioguanine selection (52). Our study of nearly 4000 *BRCA1* variant effects using genome editing likewise showed >96% concordance with established variant annotations (69). These results underscore the value relatively simple assays can have for prospective variant classification when applied to genes with well-established phenotypes.

## Searching for causal variants in non-coding sequences

Multiplex assays have been used to ask both which non-coding elements are functionally relevant and how specific variants alter function. In homage to the first saturation mutagenesis experiments studying the beta-globin promoter (70), MPRAs use reporter constructs to ask how DNA sequences function to initiate transcription. They are particularly useful for testing candidate regulatory elements nominated via association studies or biochemical annotation (71,72).

In 2009, one of the first multiplex assays to use NGS as a readout used *in vitro* transcription to test synthesized promoter fragments (73). Since then, several renditions of MPRAs have been performed using episomal- or integration-based cellular expression systems (74,75). Strategies for quantifying effects include expressed barcodes (38,39), reading out candidate elements from RNA directly (76) and using FACS to separate cell populations based on expression (77). MPRAs have also been carried out in primary cells, models of stem cell differentiation and *in vivo* (38,78–80).

Identifying causal variants in GWAS-implicated loci can provide new insights into underlying pathways that drive disease in humans (81). Once validated regulatory elements are linked to downstream gene targets, for instance, via clustered regularly interspaced short palindromic repeats (CRISPR) editing (82,83), further functional characterization may reveal drug targets (84). While there are relatively few regulatory regions known to harbor highly penetrant, pathogenic variants, MPRAs can identify which variants are critical in such regions. In one example, Doan *et al.* (85) use MPRAs to implicate homozygous variants in human-accelerated regions underlying autism cases. In an expansive effort, Kircher *et al.* (86) tested the functional effects of >30 000 point mutations across 20 non-coding regions implicated in disease, including the *TERT* and *LDRL* promoters and the *SORT1*-associated enhancer. This approach accurately identified causal variants across loci, thereby establishing the broad utility of MPRAs to aid classification of rare non-coding variants.

Multiplex assays are also showing great potential for identifying splice variants of strong effect. These assays have largely been performed using minigenes on plasmids transfected into human cell lines and have relied on transcribed barcodes, sequencing of variants from RNA or fluorescent reporter systems (56,87–90). In one powerful example, Rosenberg *et al.* (91) used splicing data from millions of degenerate sequences to train a highly accurate model for predicting splicing outcomes. A theme emerging from this work is that many splice-disruptive variants occur relatively far from canonical splice junctions, often extending deep into exons and introns. Notably, profiling >27 000 rare variants from human exomes revealed that nearly 4% disrupted splicing and that the vast majority of these occurred outside of canonical splice sites (56).

Beyond splicing, other multiplex assays to study RNA function include mutating 5′ untranslated regions (UTRs) (92) and synonymous codons (93) to study translation rates and mutating 3′ UTRs to assess messenger RNA (mRNA) stability (94). With more RNA sequencing and whole-genome sequencing being used clinically, these assays promise to illuminate additional mechanisms by which variants exert phenotypic effects in patients (95).

## Emerging themes: integration of readouts from multiple functional assays achieves greater phenotypic depth

Assays with relatively simple readouts that are broadly generalizable across loci will prove valuable for scaling experiments to meet clinical demand. However, recent studies illustrate that more phenotypically detailed information can be gained by interrogating libraries with multiple functional readouts and in multiple cell types (Table 1). Cell-based approaches in which variant libraries are stably integrated allow cells to be expanded and assayed in multiple ways (40,50,96,97). One recent study tested the same MPRA library in five different cell lines to improve the identification of causal variants from GWAS data and to nominate cell-type-specific effects (94). Using cell-based assays, a deep mutational scan of the warfarin-target VKOR was used to readout both protein stability and enzymatic activity (61). This dual approach elucidated four transmembrane domains and key active site residues, while also providing clinical insights into variants that increase warfarin sensitivity.

**Table 1:** Strategies for combining data across multiplex assays to reveal mechanisms

Strategy	Benefit	Examples
Combining readouts of protein function		
Protein stability and enzymatic activity	Corroborating pathogenicity; nominating dominant negative variants	*VKOR* (61); *PTEN* (101); nudix hydrolase 15 (*NUDT15*) (97)
Specific protein function and cell survival	Corroborating pathogenicity; linking specific functions to cell-based phenotypes	*BRCA1* (54)
Multiple drug treatments	Interrogating pathway dependencies; mapping resistance mutations	*BCR-ABL* (50); *MCL1*, *BCL2L1* (117)
Analyzing splicing and protein function		
RNA expression and cell survival	Improves clinical accuracy by identifying splice variants (including intronic)	*BRCA1* (69); *CARD11* (110)
Testing variants in multiple cell lines		
Engineered genetic backgrounds	Discerns dominant versus recessive effects; assess epistasis	*TP53* (99,100)
Different cell types	Reveals cell-type effects on gene regulation; explains mutational profiles in disease	DNA damage response pathway (118); several regulatory loci (86,94)
Cancer cell growth *in vitro* versus *in vivo*	Separating cell-intrinsic and cell-extrinsic variant effects	*TP53* (98)

Three mutational scans have been performed for *TP53*, the tumor suppressor gene most commonly mutated in human cancer. First, Kotler *et al.* (98) asked how mutations to the protein’s DNA-binding domain affect cell growth, both in culture and in tumor models. Whereas, hotspot mutations did not confer a growth advantage over null alleles *in vitro*, they did *in vivo*, a finding suggestive of potential gain-of-function effects. Meanwhile, Giacomelli *et al.* (99) leveraged CRISPR-screening data to devise assays in isogenic p53^+^ and p53-null lines using multiple drug treatments. The different combinations distinguished dominant negative variants from loss-of-function alleles. Finally, Boettcher *et al*. (100) used a leukemia line to show that hotspot *TP53* mutations act as dominant negatives, a mechanism that fully explains the *TP53* mutational landscape of acute myeloid leukemia (AML). Collectively, these papers illustrate how cellular context, genetic background and assay design can be crucial to elucidate disease mechanisms of variants.

Two groups have applied mutational scans to the tumor suppressor *PTEN*, studying variants’ effects on protein stability in human cells (55) and lipid phosphatase activity in yeast (59). Analyzing these data in conjunction with well-curated patient data revealed variants that increase autism spectrum disorder (ASD) risk and associate with early-onset cancer (101). Highlighting the value of integrating multiple assays, putative dominant negative variants were identified that retained stability but not enzymatic activity.

An alternative approach to layering multiple assays would be to use a single assay capable of capturing different functional classes of variants. Of note, one group has recently demonstrated using single-cell RNA-sequencing to interrogate patient variants observed in the oncogenes NRAS and MYC (102). Coupling expressed barcodes to each variant allowed cells to be genotyped and transcriptionally profiled, revealing distinct pathways activated by specific mutations. Though this implementation required sequencing >300 000 single cells to study 200 variants, single-cell readouts may prove advantageous for exploring variant effects with greater phenotypic depth going forward.

A common limitation to multiplex assays is the use of complementary DNA (cDNA) libraries that preclude discovery of splice-altering variants. Therefore, combining assays that assess splicing with those measuring effects on the protein level will be essential to achieve optimal clinical accuracy. Recently, >1000 variants in *POU1F1* were assayed using a minigene reporter, and 113 were deemed splice-disruptive (90). Two of these co-segregated with disease in unsolved families with familial hypopituitarism. In our work on *BRCA1*, we were able to measure variant effects on both protein function and mRNA levels by using SGE. This implicated ~10% of loss-of-function missense variants as disrupting splicing (69). With whole-genome and RNA sequencing becoming more common clinically (95,103), there will be more opportunities to link splice-disruptive variants within introns to human phenotypes using multiplex assays.

## Emerging themes: genome editing allows variants to be tested at endogenous loci with growing ease

As illustrated by variants impacting splicing, it is often advantageous to test variants at their endogenous loci. Apart from splicing, genomic context can be crucial for maintaining physiological protein expression levels and for assessing variants in regulatory elements. By way of example, a comparison of identical MPRAs performed on genome-integrated versus non-integrated constructs showed only a weak correlation in scores (104).

To our benefit, genome editing technologies have improved since the introduction of CRISPR/Cas9 to facilitate more efficient and precise editing in a wide variety of cell types (105) (Table 2). Several methods have been established to boost homology-directed repair (HDR) efficiencies, allowing more efficient integration of variants of interest (106). Haploid human lines (e.g*.* HAP1) can be edited to reveal variant effects that are recessive on the cellular level (69,107,108), though engineering polyploid cells to contain a single copy of a target locus may prove viable for more cell types (109). Likewise, a cloning-free SGE protocol was recently deployed in a diploid B cell lymphoma line to link dominant negative variants in *CARD11* to rare immunodeficiencies (110).

**Table 2:** Selected genome editing assays for testing human variant at scale

Method	Paper	Description	Assay
SGE	(40)	HDR-mediated integration of variants at Cas9-targeted loci	Hexamer effects on splicing in HEK293 (*n* = 4048); *DBR1* variant fitness in HAP1 (*n* = 365)
	(69)	(as above)	*BRCA1* variant effects on HAP1 fitness (*n* = 3893) and transcript levels (*n* = 2646)
	(110)	Cloning-free SGE with single-stranded DNA repair templates	*CARD11* variant effects on TMD8 growth +/− ibrutinib and transcript levels (*n* = 2542)
Base editor screens	(119)	gRNA libraries used with base editing to introduce specific variants	*n* = 745 gRNAs targeting all exons of *BRCA1* for fitness effects in HAP1
	(117)	(as above)	*n* = 70 000+ gRNAs tested in various cell lines (HAP1, MELJUSO, A375 and HT29) and assays (drug sensitivity, resistance and fitness of 57 000+ ClinVar variants)
	(118)	(as above)	*n* = 50 000+ gRNAs to tile 86 DNA damage response genes, assaying essentiality and response to DNA damage drugs in MCF10A, MCF7 and HAP1
Saturation prime editing	(121)	Prime editing gRNAs designed to achieve saturation mutagenesis	Variant effects on lysosome trafficking (*NPC1; n* = 256) and growth (*BRCA2*; *n* = 465) in 293T

Base editing and prime editing technologies have emerged as CRISPR-based alternatives for creating programmed variants and are continuing to improve through protein engineering (111–113). Base editors use Cas9 fused to cytosine or adenine deaminase domains to achieve targeted editing (111,114), whereas prime editing is accomplished via Cas9-directed reverse transcription to introduce programmed variants (112). One advantage of these systems is that highly specific variants are created without the need for double-stranded breaks and HDR, suggesting greater scalability may be possible (115,116).

Accordingly, the first large-scale base editor screens were recently published (117–119). Employing numerous growth-based assays in human cell lines, Hanna *et al.* assessed >52 000 ClinVar variants, discovering loss-of-function variants in disease genes and mapping protein residues where variants alter responses to targeted therapies. With similarly broad coverage, Cuella-Martin *et al.* used base editing to engineer missense variants across 86 DNA damage response genes, discovering functionally critical protein domains and providing evidence for VUS reclassification. Importantly, these screens required careful validation to confirm guide RNAs (gRNAs) scored as hits were creating the intended edits.

Though currently limited by lower editing efficiencies, optimization of prime editing systems may further facilitate saturation mutagenesis of endogenous loci (120). A recent preprint describes ‘saturation prime editing’, using libraries of prime editing gRNAs to resolve pathogenicity for hundreds of variants in *BRCA2* and *NPC1* (121). In the future, coupling improved versions of these technologies with CRISPR tools to identify regulatory elements (122) will help reveal the functional impact of rare variants at regulatory loci. Overall, the rapid pace of improvement to CRISPR reagents suggests that, in coming years, engineering large numbers of variants in their endogenous context may become as easy as engineering them on plasmids.

## Emerging themes: large genetic databases and multiplex assays synergistically improve variant classification

**Figure 2:**
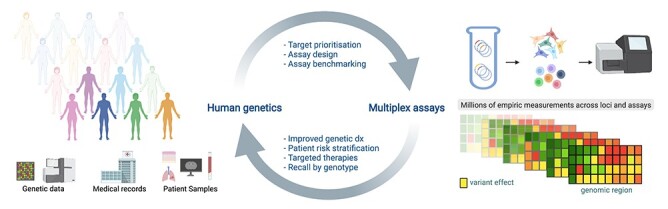
Integration of multiplex assays with genetic data from patients. Large numbers of VUS are observed in clinical testing and many loci associated with disease have yet to be functionally studied. These are priority targets to study using multiplex assays. Such assays can be rapidly validated via comparison to existing knowledge of variant effect and then integrated with large genetic data sets to improve diagnosis and help guide therapeutic strategies.

Multiplex assays are inherently orthologous to classical genetics approaches and computational predictors. Therefore, new experimental data sets can be benchmarked using established genotype–phenotype relationships. Majithia *et al.* (53) used a FACS-based assay to measure the effects of ~10 000 *PPARG* variants and found many of the lowest scoring alleles were exclusive to type 2 diabetes patients in a cohort of ~20 000 individuals. Applying SGE to *BRCA1*, we could immediately validate our results via comparison to hundreds of variant interpretations provided in ClinVar (69). Other groups have since used *BRCA1* SGE data to reanalyze variants seen in hereditary cancer predisposition testing. In one cohort, clinical records were used to show that *BRCA1* variants deemed loss-of-function by SGE confer a clinical risk indistinguishable from previously established pathogenic variants (123). This example illustrates how multiplex assays can be rapidly validated with pre-existing genetic data and subsequently used to reclassify variants observed in patients (Fig. 2).

Large genetic databases lacking disease associations (e.g*.* gnomAD) also have utility for evaluating multiplex assays. Variants predicted to be deleterious by an assay should be seen less frequently in humans if they occur in genes under purifying selection, a trend now observed across multiple studies (52,69,101). Going forward, genetic data sets with deep phenotyping, such as the UK Biobank (124), will provide greater context for linking variant effects *in vitro* to human phenotypes. Recalling patients or accessing banked samples by genotype will enable rapid follow-up studies of variants deemed functionally relevant to disease.

Variant databases are increasingly incorporating results from functional assays (17,125) as well as predictions from machine learning methods (126–128). Models of variant effect built from multiplex assays have also been used to impute missing data and to predict deleterious variants genome-wide (44,129). As more experimental data are generated, such models will incrementally gain predictive power and may soon be able to accurately predict the effects of far more variants than can be assayed currently. To maximize the benefit of both multiplex assay data and improved computational models, we will require better tools for efficiently integrating multiple lines of evidence into clinical interpretation algorithms.

## Conclusions and Future Challenges

In summary, multiplex assays have become a powerful means of generating variant effect data, and recent studies showcase how these technologies are starting to bring benefits to clinical variant interpretation.

Toward accurately reporting on the broad range of genetic effects throughout human development and disease, optimizing multiplex approaches in model systems, such as stem cells, organoids and *in vivo* models, will be a major challenge to overcome (130). Additionally, epistasis remains very difficult to test on the scale of individual variants owing to the immense number of potential interactions (131). Multiplex assays have the potential to improve variant interpretation for diverse populations that have been historically underrepresented in genetic studies (132). To make this work, however, we must ensure well-curated experimental data sets are widely available and take care to assess their utility across different populations. Global efforts to securely share genetic data will help facilitate this (133,134).

Despite these considerable challenges, the impact of these powerful technologies is already starting to be seen. By systematically testing large numbers of variants across numerous assays, we are building the basis for a more unbiased and quantitative understanding of genotype–phenotype relationships in humans. In coming years, multiplex assays will continue to reveal the genetic mechanisms underlying disease phenotypes, and in doing so, substantially improve the clinical utility of genetic data.

## Supplementary Material

Review_Table_1_Assay_Combos_ddab219

Review_Table_2_CRISPR_Strategies_ddab219
