# Supplementary material for: Linking genome variants to disease: scalable approaches to test the functional impact of human mutations
Source: Hum Mol Genet. 2021 Aug 2;30(R2):R187–97. doi: 10.1093/hmg/ddab219 (PMC8490018; doi:10.1093/hmg/ddab219)
Supplement: Review_Table_1_Assay_Combos_ddab219 [file review_table_1_assay_combos_ddab219.pdf]

Table 1. Strategies for combining data across multiplex assays to reveal mechanisms

Strategy	Benefit	Examples
**Combining readouts of protein function**		
Protein stability and enzymatic activity	Corroborating pathogenicity; nominating dominant negative variants	*VKOR* (Chiasson et al. 2020); *PTEN* (Mighell et al. 2020); *NUDT15* (Suiter et al. 2020)
Specific protein function and cell survival	Corroborating pathogenicity; linking specific functions to cell-based phenotypes	*BRCA1* (Starita et al. 2018)
Multiple drug treatments	Interrogating pathway dependencies; mapping resistance mutations	*BCR-ABL* (Ma et al. 2017); *MCL1*, *BCL2L1* (Hanna et al. 2021)
**Analysing splicing and protein function**		
RNA expression and cell survival	Improves clinical accuracy by identifying splice variants	*BRCA1* (Findlay et al. 2018); *CARD11* (Meitlis et al. 2020)
**Testing variants in multiple cell lines**		
Engineered genetic backgrounds	Discern dominant vs. recessive effects; assess epistasis	*TP53* (Giacomelli et al. 2018, Boettcher et al. 2019)
Different cell types	Reveal cell-type effects on gene regulation; explain mutational profiles in disease	several regulatory loci (Kircher et al. 2019, Griesemer et al. 2021)
Cancer cell growth *in vitro* vs *in vivo*	Separating cell-intrinsic and cell-extrinsic variant effects	*TP53* (Kotler et al. 2018)
