# Supplementary material for: Linking genome variants to disease: scalable approaches to test the functional impact of human mutations
Source: Hum Mol Genet. 2021 Aug 2;30(R2):R187–97. doi: 10.1093/hmg/ddab219 (PMC8490018; doi:10.1093/hmg/ddab219)
Supplement: Review_Table_2_CRISPR_Strategies_ddab219 [file review_table_2_crispr_strategies_ddab219.pdf]

Table 2. Selected genome editing assays for testing human variants at scale

Method	Paper	Description	Assay
**Saturation genome editing**	Findlay et al. (2014)	HDR-mediated integration of variants at Cas9-targeted loci	Hexamer effects on splicing in HEK293 (n = 4,048); *DBR1* variant fitness in HAP1 (n = 365)
	Findlay et al. (2018)	"	*BRCA1* variant effects on HAP1 fitness (n = 3,893) and transcript levels (n = 2,646)
	Meitlis et al. (2020)	cloning-free SGE with single-stranded DNA repair templates	*CARD11* variant effects on TMD8 growth +/- ibrutinib and transcript levels (n = 2,542)
**Base editor screens**	Kweon et al. (2020)	gRNA libraries used with base editing to introduce specific variants	n = 745 gRNAs targeting all exons of *BRCA1* for fitness effects in HAP1
	Hanna et al. (2021)	"	n = 70,000+ gRNAs tested in various cell lines (HAP1, MELJUSO, A375, HT29) and assays (drug sensitivity, resistance, fitness of 57,000+ ClinVar variants)
	Cuella-Martin et al. (2021)	"	n = 50,000+ gRNAs to tile 86 DNA damage response genes, assaying essentiality and response to DNA damage drugs in MCF10A, MCF7 and HAP1
**Saturation prime editing**	Erwood et al. (2021)	prime editing gRNAs designed to achieve saturation mutagenesis	Variant effects on lysosome trafficking (*NPC1;* n = 256) and growth (*BRCA2;* n = 465) in 293T
